# Fundamental patterns and predictions of event size distributions in modern wars and terrorist campaigns

**DOI:** 10.1371/journal.pone.0204639

**Published:** 2018-10-17

**Authors:** Michael Spagat, Neil F. Johnson, Stijn van Weezel

**Affiliations:** 1 Department of Economics/Royal Holloway University of London, Egham, Surrey, United Kingdom; 2 Physics Department, George Washington University, Washington D.C. 20052, United States of America; 3 Unaffiliated, Frankfurt am Main, Germany; University of Essex, UNITED KINGDOM

## Abstract

It is still unknown whether there is some deep structure to modern wars and terrorist campaigns that could, for example, enable reliable prediction of future patterns of violent events. Recent war research focuses on size distributions of violent events, with size defined by the number of people killed in each event. Event size distributions within previously available datasets, for both armed conflicts and for global terrorism as a whole, exhibit extraordinary regularities that transcend specifics of time and place. These distributions have been well modelled by a narrow range of power laws that are, in turn, supported by some theories of violent group dynamics. We show that the predicted event-size patterns emerge broadly in a mass of new event data covering all conflicts in the world from 1989 to 2016. Moreover, there are similar regularities in the events generated by individual terrorist organizations, 1998—2016. The existence of such robust empirical patterns hints at the predictability of size distributions of violent events in future wars. We pursue this prospect using split-sample techniques that help us to make useful out-of-sample predictions. Power-law-based prediction systems outperform lognormal-based systems. We conclude that there is indeed evidence from the existing data that fundamental patterns do exist, and that these can allow prediction of size distribution of events in modern wars and terrorist campaigns.

## Introduction

Polymath Lewis Fry Richardson showed, in his seminal work, that war sizes follow a fat-tailed distribution which, he suggested, could be well captured by a power law [1, 2]. Later research has updated and confirmed this finding using more rigorous statistical methods [3–5]. It turns out that the Richardson insight for sizes of whole wars extends to event sizes *within* wars. For this analysis the size of a discrete event, such as a suicide bombing or a battle, is defined by the number of people killed in the event. The distributions of event sizes within nine modern wars are all well approximated by a power law with the estimated power coefficients clustering around 2.5 [6]. The size distribution for global terrorist events, merging together all events perpetrated by all terrorist groups, is also well captured by a power law with a coefficient around 2.5 [7]. This latter finding has practical utility because the identified empirical regularities can be used to predict the probability of a terrorist attack comparable in scale to the 9/11 one [8, 9].

The present paper has three main objectives. First, we exploit a mass of new event data on armed conflict and terrorism [10, 11] to offer the most complete exploration ever presented of the empirical patterns in the size distributions of violent events within the contexts of both armed conflict and terrorist campaigns. For our conflict analysis we use the new version of the data employed in previous research [12], enabling us to extend our reach to no fewer than 273 armed conflicts, including more than 100 Asian conflicts never before included in this research program. Our empirical work on terrorism innovates by operating at the *organization* level, enabling us to demonstrate that the size distributions of violent events perpetrated by 60 individual terrorist organizations resemble the size distributions we find for belligerent groups entangled in armed conflicts. This finding deepens an already identified link between terrorist and insurgent organizations [6], which is reassuring given the notorious difficulty in separating the two types of organizations conceptually [13]. Indeed, although it may be possible to draw valid distinctions between insurgent versus terrorist organizations, e.g., concerning their ideologies, they both remain collections of decentralized operatives that must adapt quickly to avoid detection and annihilation [14]. These common pressures should force both types of groups into common David-versus-Goliath tactics that should yield similar attacking patterns and, indeed, we find this in our empirical work. Note, however, that we only include terrorist campaigns that survive long enough to make at least thirty attacks. Such persistent terrorist organizations are the best candidates to resemble insurgencies.

Second, we explore the empirical robustness of the event-size patterns we find in our 273 armed conflicts. Ref. [6] found strongly consistent patterns in 9 modern wars and global terrorism but the scope of that study is dwarfed by the present one which covers all conflicts in the world since 1989 with at least 30 events. So there remains an open empirical question: do all modern conflicts share some deep structure that transcend specific characteristics of individual conflicts and that expresses itself in largely common event-size patterns? We tackle this question by testing our ability to make reasonably accurate predictions about the mixtures of event sizes in randomly chosen conflicts, based only on the range of empirical power-law coefficients we have observed in entirely different conflicts. We find that we can, indeed, make accurate out-of-sample predictions. The main conclusion we draw from this predictability in event-size patterns is that there is a deep underlying structure for modern conflicts that remains stable from conflict to conflict. A practical implication of this finding is the insight that even conflicts that have generated only small events for long periods of time are likely to still retain a latent potential to eventually generate some very large event sizes. Indeed, there is a maximum event size of 20 in 50 of our 273 conflicts, 17 of which were still active in 2016 including in Kenya, Thailand, Myanmar, the Philippines, ISIS activity in Russia, and drug cartels in Mexico: these countries should prepare to potentially suffer some much larger events.

Third, we test the predictive performance of the power-law representation of armed conflict event sizes against that of a lognormal-based system, the most obvious fat-tailed rival distribution. We find that power-law based prediction systems outperform lognormal ones, confirming the value of power laws for modelling event-size distributions for armed conflict and terrorist campaigns.

It is worth pausing for a moment to clarify two potentially confusing points. First, we pursue a rather non-standard prediction agenda. For example, we never predict that an event of size *x* will occur with some probability within some spatial or temporal interval. Rather, we predict broad event-size patterns within known conflicts based only on data from other conflicts. We do this in pursuit of fundamental knowledge about how modern conflicts work. The predictability in event-size patterns we find in all significant conflicts reaching back to 1989 presents a theoretical challenge; how can we explain the underlying consistency that ranges across so many conflicts? A more standard prediction agenda aims to provide directly actionable intelligence, e.g., that a particular country is at risk of slipping into war or political instability in a particular year [15]. That said, we think there is some practical utility to our main finding that the size distribution of events in randomly chosen modern conflicts can be well predicted by a particular range of power laws because this suggests that all modern conflicts retain a latent potential to generate very large events even if they have only produced relatively small ones for a long time. In other words, there seems to be no such thing as a modern conflict that can only generate small events. Conflict-torn countries should prepare for big events.

Second, one might reasonably ask how surprising our results are given that Ref. [6] had similar findings and predicted that all modern wars would greatly resemble the 9 wars analyzed in that paper. The answer is that Ref. [6] made a prediction which we are now largely confirming, subject to some variation, based on data from all conflicts since 1989 with at least 30 events, 273 in all.

## Materials and methods

We take our armed-conflict data from the Georeferenced Event Dataset (GED) of the Uppsala Conflict Data Programme [10]. This is the most comprehensive and accurate georeferenced dataset on armed conflict available [16–18] that systematically collects information on the number of people killed in each event. The GED records details that include the location, timing, and severity of conflict events along with information on the warring parties that generate these events. The data collection effort covers conflicts between governments and rebel groups, non-state based conflicts (also known as communal violence), and violence perpetrated by the state or insurgency groups against civilians. We use the most recent version of the dataset available at time of writing (v.17.2) which covers all conflicts across the globe between 1989-2016. The GED coding rules exclude some low-intensity conflicts by imposing a minimum fatality threshold of 25-battle related deaths in a given year. However, this restriction hardly matters for us since it excludes only minor conflicts that may have been excluded anyway due to not having enough events to allow us to reliably fit a power law to the size distribution of their violent events.

We include only true single events in our analysis, removing a number of aggregate fatality counts that are not broken down to the event level; and we also drop conflicts with fewer than 30 events, based on the included conflict identifier. The first restriction is necessary so that we work with true event data without mixing in items that appear to be very large events but which are really composites of multiple smaller events. We fit a power law to every conflict so something along the lines of the second restriction is also necessary to avoid fitting power laws to just a handful of data points. If anything, we should require a larger number of events such as 50 or 100 to get better fits than we do to individual conflicts but this would be at the cost of excluding more conflicts from our analysis. The two restrictions combined mean that of the original 997 conflict in the dataset, with 135,181 events, we are left with 272 conflicts and 105,685 events, or 78% of the original events. For Afghanistan we split the state-based data into two separate conflicts so that the fighting after the beginning of Operation Enduring Freedom is treated separately from the pre-invasion conflict, bringing our total count up to 273 conflicts.

We offer a parallel analysis of terrorist incidents since, as noted above, there is evidence suggesting that terrorist organizations may behave similarly to insurgent groups [6, 19, 20]. For this work we use the Global Terrorism Database (GTD, the 2017 version), which is provided by the National Consortium for the Study of Terrorism and Responses to Terrorism (START). A novel feature of the GTD dataset is that it includes both domestic and trans/inter-national terrorist incidents. The GTD is updated annually and provides the most comprehensive dataset on terrorist events that is publicly available, covering the period from 1970 to 2016 and including detailed information on incident times, locations, fatality counts and, when identifiable, the perpetrating group or individual. We include only events that are, according to the coding, definitely acts of terrorism causing at least one fatality and that are attributed to a known organization that has perpetrated at least 30 attacks. Finally, we use only events occurring after 1997 because the GTD coding procedures changed in that year. This leaves us with 16,399 terrorist attacks carried out by 60 groups between 1998-2016.

## Results

**Fig 1:**
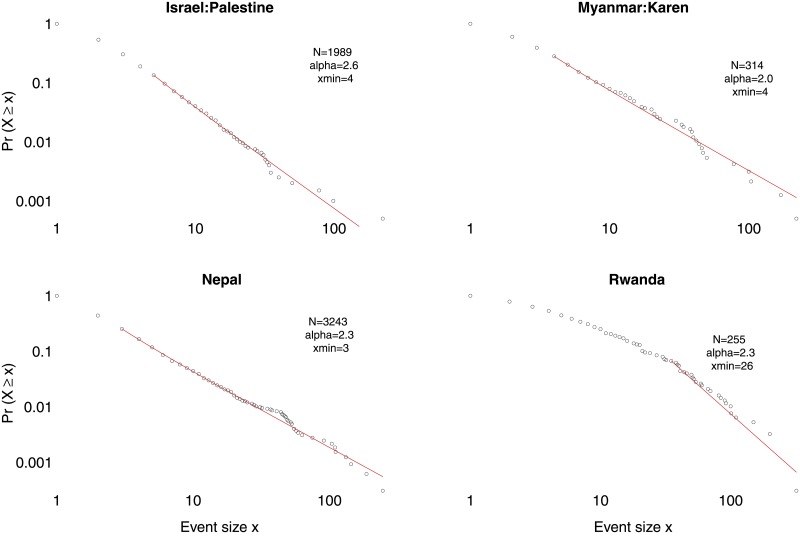
Fitted power laws for four conflicts. Event sizes measured as number of people killed are plotted on the *x*-axis against the fraction of events of that size or larger on the *y* axis. Red solid lines indicate fitted power laws. *Data*: UCDP-GED.

**Fig 2:**
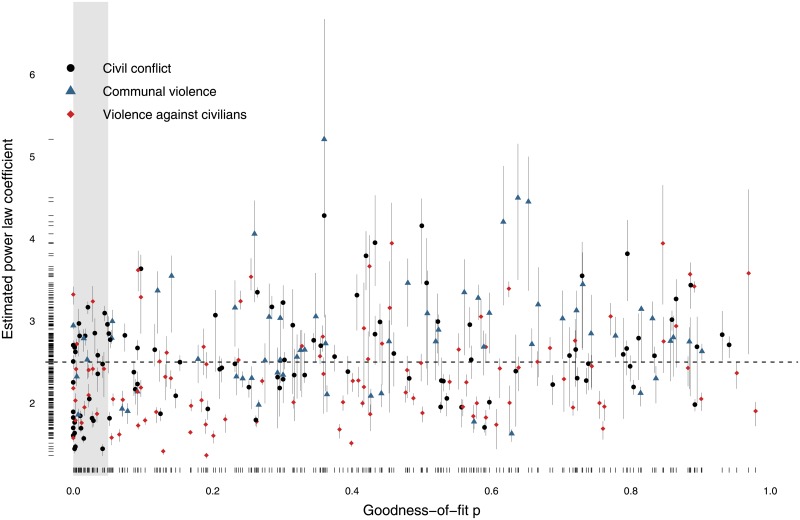
Estimates of *α* parameter, along with 50% uncertainty interval, versus *p* values for power-law hypotheses for global violent armed conflicts. Grey shaded area corresponds to goodness-of-fit *p* ≤ 0.05.

We use the “poweRlaw” package in R [21] to fit the model *Ms*^−*α*^ to the data for each conflict above an estimated cut-off value *s*_*min*_ using maximum likelihood estimation [12, 22] where *s* denotes the number of fatalities in an event, *α* is the power-law coefficient and *M* is a normalisation factor ensuring that the cumulative probability distribution sums to unity. Fig 1 provides examples of power laws fitted to four conflicts, The *s*_*min*_ parameter, the lower threshold, is estimated using a Kolmogorov-Smirnov approach, where the distance between the cumulative density function of the data and the fitted model is minimised [21, 22]. To account for parameter uncertainty, the estimates are obtained using a bootstrap procedure with 1,000 iterations. Fig 2 plots the estimated *α* values for the 273 conflicts in our sample against the *p*-values of bootstrapped tests of the hypotheses that their data are generated by the fitted power laws for these conflicts; here the null hypothesis is that the power law distribution cannot be ruled out. To be clear, each data point in Fig 2 summarizes a power law fit for one particular conflict such as each one of the conflicts in Fig 1.

Most conflicts do have size distributions for their violent events that are well fit by power laws with coefficients clustering around 2.5. At the same time, some conflicts do display *α* values far from 2.5. Moreover, some conflicts have very low *p*-values, thereby deviating from the empirically and theoretically grounded patterns uncovered in earlier research [6, 8, 9] by suggesting that the power-law hypothesis should be rejected. Low *p*-values are not necessarily a serious worry since no distribution of violent conflict events will be, literally, generated by an exact power law so we would normally expect to reject the power-law hypothesis with enough data even when this distribution is still useful for modelling the event-generating process of a conflict. Estimated *α*’s far from 2.5, on the other hand, are a more important challenge to the received wisdom in the field. These results could stem from data problems, e.g., not having enough data or having serious flaws in the data-gathering processes for particular conflicts. In fact, in earlier research [12] the conflict in Angola had a very high value of *α* but now, with a few more years’ worth of data, Angola’s *α* has settled in at 2.2. Or it could be that some modern conflicts really are fundamentally different from the great majority of conflicts we have encountered so far in this research program.

**Fig 3:**
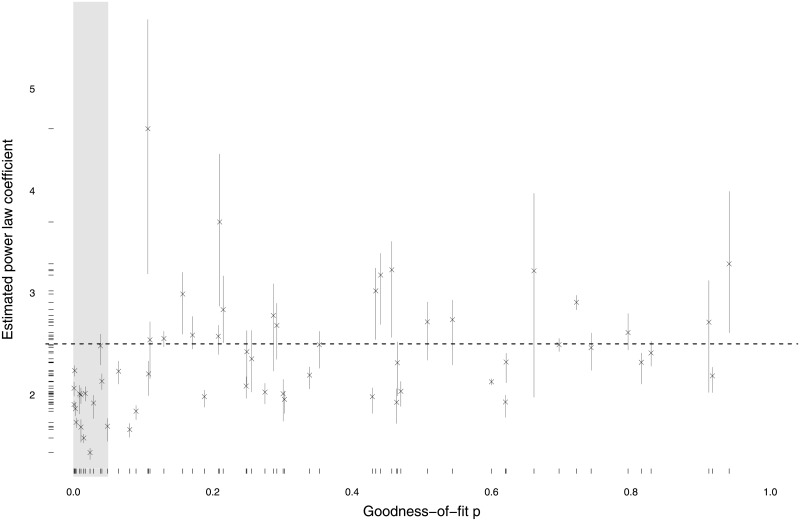
Estimates of *α* parameters versus *p* values for power-law hypotheses for terrorist organizations. Grey shaded area corresponds to goodness-of-fit *p* ≤ 0.05. *Data*: GTD.

Fig 3 provides the same sort of *p* versus *α* information given in Fig 2 but this time for terrorist groups using the GTD data. Note that the nature of these results is substantially different from earlier work fitting power laws to global terrorist events [7] because we fit a separate power law to each terrorist organization whereas the previous work merged together all the events generated by all terrorist organizations. It shows that power laws with *α* values that cluster around 2.5 also tend to fit well the distributions of violent events generated by terrorist organizations. Thus, there appear to be close parallels in the behavior of terrorist and insurgent organizations, at least with respect to the processes that generate their violent events. This empirical commonality is reassuring given the blurred distinctions between the two types of organizations [13].

We now exploit the empirical findings displayed in Fig 2 to make predictions about event-size patterns. For clarity, recall that we do not make definitive and specific statements about the timings and locations of a conflict events (point prediction) or probabilistic statements covering longer time periods (forecast) [23]. Predicting armed conflict in these senses is extremely difficult, although potentially worthwhile if it can be done well. Rather, we focus on predictions over broad patterns [24], a task that is, perhaps, easier than point prediction and forecast although still a great challenge.

For the cross-validation we generate out-of-sample predictions for the expected ratios of event counts for various pairs of size ranges and then calculate the successes and failures of these predictions. Specifically, we implement the following procedures.

1. Randomly split the sample into two parts and use one third of the conflicts to generate out-of-sample predictions for the remaining two thirds of the conflicts.
2. Fit power laws to the third that was selected.
3. Order the *α* estimates from the selected third from smallest to largest and calculate the range of *α*’s running from percentile 2.5 to percentile 97.5.
4. Use the lower and upper bounds of this range to predict the upper and lower bounds, respectively, of the ratios of event-size counts for various ratios of event size ranges.
  - For example, if the lower bound for *α* is 2.0 then the upper bound for the ratio of the number of events of size *S* or greater to the number of events of size 2*S* or greater is 2 while if the upper bound for *α* is 3.5 then the lower bound for the same ratio of event-size ranges is about 5.7*S*. The corresponding figures for *S* and 1.5*S* are 1.5*S* and 2.8*S* respectively.
5. Check these predictions against the data for the two thirds of conflicts that were not randomly selected. Although we could check a near-endless list of predictions we confine ourselves to just checking the ratios for which we multiply the event size by either 1.5 or 2.0.
6. Start over, taking a new draw of 1/3 of the conflicts and again testing the out-of-sample predictions on the remaining 2/3 of conflicts.

**Fig 4:**
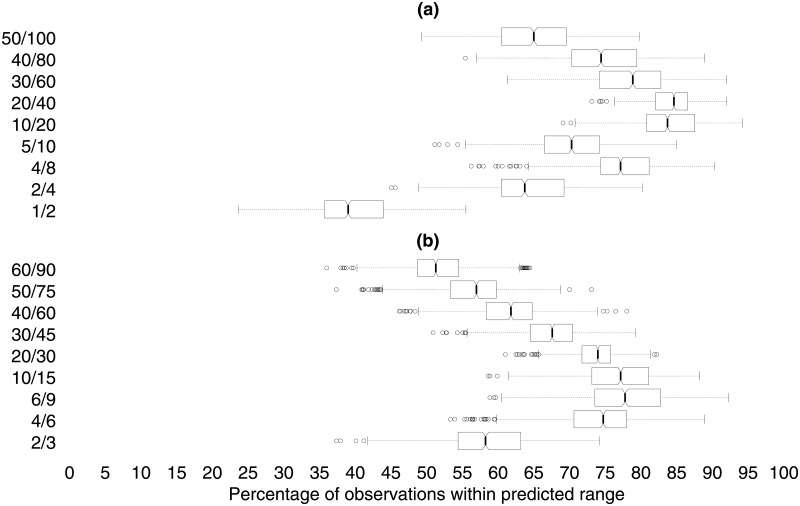
Boxplots for the distributions of the percentage of successful out-of-sample predictions for a variety of ratios of event-size ranges.

We repeat this procedure 1,000 times. Fig 4 displays the results for this simulation exercise. For most event-size ratios the success rates exceed 60% for at least 75% of the draws of 1,000. The best prediction performance is for the event-size ratios of 10/20 and 20/40 for which the median success rates are in the 80’s and even the worst runs tend to score well above 60%. The worst prediction performances are when the events are either very small or very large. The relatively low success rates for small events make sense since the estimated power laws are not even meant to apply below some cut-off level *s*_*min*_. Thus, if anything, the success rates for the low-end predictions are a bit of a bonus. Relatively weak performance at the high end also makes sense since the data on big events are sparse, providing only a thin empirical basis for prediction. Note, further, that these good prediction scores are not generally due to vacuously wide prediction intervals as the typical intervals are around 1.2 to 3.5 and 1.4 to 8.6 for size ranges of the form *S* to 1.5*S* and *S* to 2*S* respectively (with the upper limit of 8.6, admittedly, rather high).

**Fig 5:**
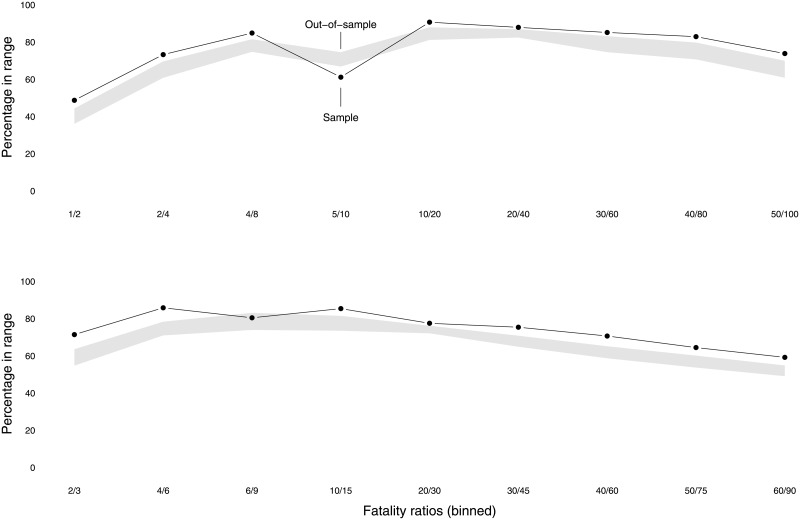
The success rates for in-sample predictions compared to the success rates for out-of-sample predictions. The shaded area indicates the 50% interval for the out-of-sample results.

Fig 5 shows that out-of-sample prediction works almost as well as in-sample prediction for our power-law based scheme. The solid curves give the success rates when we use all data to generate the *α* range and then test the predictions (self-referentially) on the whole dataset. The grey-shaded area indicates the middle 50% of the success rates for the 1,000 out-of-sample runs.

**Fig 6:**
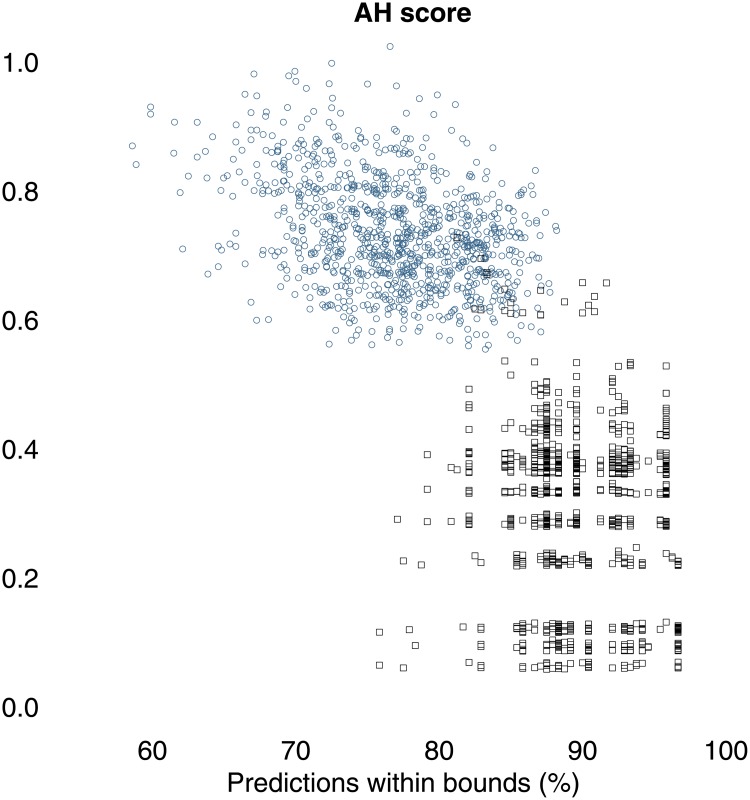
Prediction intervals for the power-law-based prediction system tend to be narrower than the prediction intervals based on the lognormal model at little or no cost to prediction accuracy. Blue circules are for power-law based predictions while black squares are for lognormal based predictions.

Next we compare the performance of our power-law-based predictions with a similar scheme that uses the lognormal distribution instead. Each point in Fig 6 gives two statistics describing the outcome of out-of-sample predictions for a particular randomly split sample. The *x*-axes give the percentages of within-boundary predictions for the 10/15 ratio, ranging over all the out-of-sample conflicts. The *y*-axes provide a measure that combine considerations of how accurate and how unhedged, i.e., how narrow, each prediction interval is. Specifically, we define the Accuracy Hedging score (*AH score*) for a prediction interval as the inverse of the root mean squared distance from its boundary predictions (percentiles 2.5 and 97.5) to the actual 10/15 fatalities ratio. Thus, the AH score most strongly rewards prediction intervals that are both accurate, i.e., centered around the true value, and minimally hedged, i.e., narrow. Fig 6 shows the power law system beating the lognormal system: the power-law based prediction intervals have systematically higher AH scores than the lognormal-based intervals do with little or no cost to the percentage of correctly predicted ratios.

Our success at out-of-sample predictions based on a range of power-law models indicates that our approach should work well for predicting the mixtures of event sizes in future armed conflicts. Both the median and the mean *α* for our 273 power-law fits is 2.5 so this is the single best rule-of-thumb value to use for such predictions. Ordering the 273 *α*’s from smallest to largest we find that an *α* of 1.6 stands at percentile 2.5 and an *α* of 4.0 is at percentile 97.5. Thus, we can predict that for any *s* the ratio of the number of events of size *s* or more to the number of events of size 1.5*s* or more will be approximately 1.8 with a prediction interval of 1.3 to 3.4. For events of sizes *s* and 2*s* the analogous numbers are 2.8, 1.5 and 8.0. It is worth noting that in the working paper version of the present paper we had data only through 2014 and found a marginally wider range for *α* running from 1.5 to 4.1 rather than from 1.6 to 4.0 [25].

## Conclusion

We have investigated the size distribution of violent events in modern conflicts and terrorist campaigns, finding that these are generally well approximated by power laws with *α* coefficients clustered near 2.5. There are some exceptions, though Figs 2 and 3 show that these exceptions also tend to have large uncertainties in the values of their coefficients. It will be interesting in the future to look in detail at what might make these few conflicts and campaigns so different. We exploit these empirical regularities in the conflict data, without ignoring the anomalies, and are able to make good predictions about the relative frequencies of violent events falling within various size classes. Our success at out-of-sample predictions indicates that our approach should work well for predicting the mixtures of event sizes in future armed conflicts. We recommend using an *α* near 2.5 for making such predictions, with a possible range of 1.6 to 4.0.

We stress once again that the main value of our findings is the fundamental knowledge about modern conflicts that they afford. At the same time there is practical value in knowing that modern conflicts do have a tendency to generate very large events if they continue long enough. We can use our prediction ranges to quantify this observation. For example, we can say that the number of events in which at least 80 people are killed is rather unlikely to be smaller than 1/8 the number of events in which at least 40 people are killed.

In the future, one could use the empirical findings in this paper concerning the power-law testing and exponent, as a way of evaluating the appropriateness of models that seek a generative, minimalistic explanation of human conflict. Among these, is a coalescence-fragmentation model originally proposed in Ref. [6] in which two populations fight, and which takes into account the tendency of clusters of insurgents to assemble for clashes or attacks and then disperse afterwards. It was shown in Ref. [6] that this two-population model gives very good agreement for the entire distribution of casualties in various conflicts—not just the approximate power-law tail, but also the low-casualty and high-casualty deviations. If one is interested only in examining the tail of the distribution, as we do in the present paper, then a simpler version of this theory is possible in which the dynamical clustering is treated as a stochastic noise term. In this case, the effect of this co-existing coalescence and fragmentation of clusters of fighters produces a distribution of cluster sizes that has a robust power-law form with a mathematically derivable exponent of 2.5. Taking the size of a cluster as a measure of its potential for damage, this suggests that the distribution of casualties should also be a power-law with exponent around 2.5. As first explained in 2005 [26], this model corresponds to the picture that an organization’s total attack strength *N* is continually being re-partitioned through coalescence and fragmentation events. The value of this attack strength *N* derives from the number of its members, its weaponry, its information etc. and hence does not lead to the conclusion that the size of the organization bounds the severity in any way. We know of no other model that has such a plausible microscopic mechanism, and yet which also predicts a clustering of power-law exponents around 2.5 without specifically cherry-picking model parameter values. Generalizing the model to allow for larger or smaller clusters to be even more rigid or more fragile than expected, yields a prediction that the casualty distribution should be power-law-like with an exponent ranging from approximately 1.5 to approximately 3.5 [12]. This is broadly consistent with our findings that place this range between 1.6 (percentile 2.5) and 4.0 (percentile 97.5).

Resolving exactly what mechanisms such a generative model should include, would of course require observing the inner workings of necessarily secretive insurgencies and terrorist organizations—which is practically impossible. Yet there is direct evidence that online ISIS communities display the coalescence and fragmentation behaviors that are central to this coalescence-fragmentation model [27]. We note that other models have been proposed, in particular by Ref. [7], Ref. [28], and Ref. [14]. However the fact that the spread of empirical values seem to be centered around 2.5, is not explained by these other models. For example, the model of Ref. [7] predicts any exponent value of 2 or larger with a priori equal likelihood, which is not what we observe.

These results also deepen our understanding of the connections between terrorism and modern warfare. These apparently different phenomena display deep common patterns that transcend their surface-level differences. Analysts of modern war and terrorism have been correct to broadly view these contentious situations as archetypal David versus Goliath confrontations [13, 19, 20].
